# Eight-Year Survival Analysis of Patients With Dilated Cardiomyopathy: Does Treatment Era Affect Prognosis?

**DOI:** 10.7759/cureus.87586

**Published:** 2025-07-09

**Authors:** Lukas Kucera, Martin Chudý, Marcela Danková, Eva Goncalvesová

**Affiliations:** 1 Department of Heart Failure and Heart Transplantation, Faculty of Medicine, Comenius University, Bratislava, SVK

**Keywords:** comorbidities, dilated cardiomyopathy, heart failure, heart transplantation, long-term prognosis

## Abstract

Background

Dilated cardiomyopathy (DCM) is a leading cause of heart failure (HF). We retrospectively analyzed long-term survival in DCM and the impact of clinical factors on their prognosis.

Methods

This was a retrospective analysis of 622 DCM patients (484 men, 138 women). Survival was compared between the 2016-2019 and 2020-2023 cohorts.

Results

The mean age was similar between cohorts (54 ± 13 vs. 55 ± 13 years). Mean overall survival for the entire cohort was 84.1 ± 1.6 months (95% CI: 81.0-87.4). When analyzed by period, mean survival was 84.1 ± 2.0 months (95% CI: 80.3-87.9) for patients diagnosed between 2016 and 2019 and 53.4 ± 1.1 months (95% CI: 51.2-55.6) for those diagnosed between 2020 and 2023. The difference was not statistically significant (log-rank p = 0.856). The shorter mean survival in the later period reflects the limited follow-up time due to ongoing observation.

In the 2020-2023 group, a higher proportion of patients were classified as New York Heart Association (NYHA) III/IV (56% vs. 48%, p = 0.036) and had larger ventricular diameters (left ventricular end-diastolic diameter (LVEDD): 68 ± 8 mm vs. 66 ± 7 mm, p = 0.001; right ventricle (RV): 36 ± 7 mm vs. 34 ± 6 mm, p = 0.001). Treatment with sodium-glucose cotransporter-2 inhibitors (SGLT2i) did not significantly affect survival. Multivariable analysis identified older age, NYHA class III/IV, chronic kidney disease (CKD) stages 3-5, diabetes, and N-terminal pro-B-type natriuretic peptide (NT-proBNP) >3000 ng/L as independent negative predictors, while female sex and overweight status were associated with better survival. Multivariable analysis identified older age, NYHA III/IV, CKD stages 3-5, diabetes, and NT-proBNP >3000 ng/L as independent negative predictors. Female sex and overweight status were associated with improved survival.

Conclusions

Survival in DCM patients remained stable across time periods, despite a higher-risk profile in recent years, potentially influenced by the COVID-19 pandemic.

## Introduction

Heart failure (HF) is a complex clinical syndrome most commonly caused by coronary artery disease (CAD), hypertension, valvular heart disease, diabetes mellitus (DM), and cardiomyopathies such as dilated cardiomyopathy (DCM) [1]. In recent years, HIV has become a notable factor contributing to the development of HF [2]. DCM currently represents the second most frequent HF phenotype and is a major indication for heart transplantation, following ischemic heart disease [3]. DCM is defined as the presence of left ventricular (LV) dilation and global or regional systolic dysfunction that cannot be explained by abnormal preload or afterload, nor by direct myocardial loss in the context of CAD [4]. Today, DCM is an umbrella term encompassing various pathological processes and genotype-environment interactions that result in a common phenotype of a dilated, dysfunctional LV [5]. New drugs and devices have helped improve survival for those living with DCM. The average 10-year survival rate was reported in an unselected population of DCM patients diagnosed since 2005, with a mean age of 52 years [6]. The epidemiology of DCM remains insufficiently explored, partly due to diagnostic challenges and the dominant manifestation, which can lead to an initial diagnosis of an otherwise unrecognized cardiomyopathy (e.g., arrhythmias, thromboembolism, or incidental CAD). Recent surveys suggest that the prevalence of DCM is approximately 118.3 individuals per 100,000 population, with idiopathic DCM accounting for around 59.2 per 100,000 [7]. The etiology and clinical course of DCM are highly variable, as is the response to treatment. Beyond classical risk factors, social determinants of health are increasingly recognized as significant contributors to HF risk [8]. Recent studies have shown significant changes in HF management over the past decade, including the introduction of novel pharmacotherapies and the potential impact of the COVID-19 pandemic on patient outcomes [9,10]. Therefore, a retrospective analysis comparing different treatment eras is justified. The aim of this analysis is to describe the characteristics and survival of patients with DCM managed at a single tertiary center, compare survival across two time periods, and evaluate risk factors affecting the survival of patients with DCM. The aim of this analysis is to describe the characteristics and survival of patients with DCM managed at a single tertiary center, compare survival across two time periods, and evaluate risk factors affecting the survival of patients with DCM.

## Materials and methods

Patients and methods

A retrospective analysis was conducted on medical records of patients admitted to a tertiary cardiac center offering advanced HF treatment options, with a diagnosis of DCM between January 1, 2016, and December 31, 2023. Data were collected from the hospital information system. The study included both patients with newly diagnosed HF and those with a known diagnosis of DCM referred for evaluation of advanced HF therapies. This retrospective design was chosen to allow the assessment of real-world trends in patient characteristics, treatment strategies, and outcomes over time, using comprehensive data from a large, unselected patient population. The two time periods (2016-2019 and 2020-2023) were chosen to reflect changes in diagnostic and therapeutic approaches in DCM management, to assess whether the COVID-19 pandemic had an impact on patient survival during this era and also the implementation of novel pharmacological therapies (e.g., sodium-glucose cotransporter-2 inhibitors (SGLT2i)).

All patients underwent a comprehensive, protocol-based diagnostic workup, including clinical examination and laboratory testing (creatinine, electrolytes, fasting glucose, lipid profile, liver enzymes, high-sensitivity troponin T, N-terminal pro-brain natriuretic peptide (NT-proBNP), C-reactive protein, and complete blood count), as well as electrocardiography (ECG), echocardiography, and coronary angiography [11]. Echocardiographic examinations were performed by experienced cardiologists. DCM was defined as LV dilatation and systolic dysfunction not explained solely by abnormal loading conditions (such as hypertension, valvular disease, or congenital heart disease) or by significant CAD [4]. Patients were included if they met both of the following criteria: first-time hospitalization at a tertiary cardiac center with a diagnosis of DCM and hospitalization occurring within the defined study period. Patients were excluded if they had any identifiable alternative cause of HF.

A total of 622 patients with DCM were included in the analysis, consisting of 484 men and 138 women. Patients were divided into two groups based on the period of hospitalization: the first group included patients hospitalized between January 1, 2016, and December 31, 2019, and the second group included those hospitalized between January 1, 2020, and December 31, 2023. Data on patient survival were obtained from the government database. In the study population, the date of the first hospitalization at a tertiary center was considered the start of follow-up, and death, the need for mechanical circulatory support (MCS), or heart transplantation (HTx) were considered final events. The date of February 2, 2025, was selected as the end of follow-up. We recorded the number of patients with an implantable cardioverter defibrillator (ICD) and cardiac resynchronization therapy (CRT) at the time of initial discharge and at the end of follow-up. 

Patients were managed according to the contemporary guidelines of the European Society of Cardiology (ESC) for the treatment of HF and the availability of specific treatment modalities in the country during the time period of their treatment [1,12]. Medication doses were titrated to the maximum tolerated levels prior to discharge. Further dose adjustments and ongoing management were performed in collaboration with the referring cardiologist. In this analysis, we report the medication use and dosages at the time of initial hospital discharge.

Statistical analysis

All statistical analyses were conducted using IBM SPSS software version 20.0 (IBM Corp., Armonk, NY). Quantitative variables were analyzed using descriptive statistics, including the number of measurements, mean, and standard deviation. Qualitative variables were analyzed using absolute and relative frequencies. Differences between groups for quantitative variables were tested using an independent samples t-test or analysis of variance (ANOVA), and for qualitative variables using the chi-square test of independence. Mean and median survival times were estimated using the Kaplan-Meier method, with 95% confidence intervals provided for each group. Comparisons between subgroups were evaluated using the log-rank test. Univariate and multivariate Cox proportional hazards model (hazard ratio (HR)) was used to identify independent prognostic factors. The level of statistical significance was set at α = 0.05.

## Results

Baseline characteristics

The study population consisted of 622 patients diagnosed with DCM. Patients were referred to our tertiary center either for further management of their condition or for evaluation of advanced HF treatment options. The time interval between initial diagnosis and referral to the tertiary center was not assessed. Baseline characteristics of the study population are presented in Table 1.

**Table 1:** Baseline characteristics of the study cohort ACEI - angiotensin-converting enzyme inhibitor; AF - atrial fibrillation; ALT - alanine aminotransferase; ARB - angiotensin receptor blocker; ARNI - angiotensin receptor/neprilysin inhibitor; AST - aspartate aminotransferase; BB - beta-blockers; BIL-T - total bilirubin; BMI - body mass index; BPM - beats per minute; Chol - cholesterol; CKD - chronic kidney disease; CKD-EPI - Chronic Kidney Disease Epidemiology Collaboration; CRT - cardiac resynchronization therapy; DBP - diastolic blood pressure; DCM - dilated cardiomyopathy; HGB - hemoglobin; HDL-C - high-density lipoprotein cholesterol; ICD - implantable cardioverter defibrillator; IVS - interventricular septum; LA - left atrium; LDL - low-density lipoprotein cholesterol; LVEDD - left ventricular end diastolic diameter; LVEF - left ventricular ejection fraction; MR - mitral regurgitation; MRA - mineralocorticoid receptor antagonists; n - number; NT-proBNP - N-terminal pro-B-type natriuretic peptide; NYHA - New York Heart Association; PLT - platelets; PWD - posterior wall diameter; RV - right ventricle; SBP - systolic blood pressure; SGLT2i - sodium-glucose transport protein 2 inhibitors; TAPSE - tricuspid annular plane systolic excursion; TG - triglycerides; TR - tricuspid regurgitation; WBC - white blood cells *The level of statistical significance was set at α = 0.05. Qualitative variables were analyzed using absolute and relative frequencies. Differences between groups for quantitative variables were tested using an independent samples t-test or analysis of variance (ANOVA). Differences between groups for qualitative variables were tested using the chi-square test of independence, except for the variable “died during follow-up,” which was compared using the log-rank test to account for unequal follow-up duration.

Patient characteristics	All patients	First time period (January 1, 2016, and December 31, 2019)	Second time period (January 1, 2020, and December 31, 2023)	p-value
Male n (%)	484 (78.0)	258 (76.0)	226 (80.0)	0.154
Female n (%)	138 (22.0)	83 (24.0)	55 (20.0)	
Died during follow-up n (%)	155 (24.9)	115 (33.7)	40 (14.2)	0.706*
Age (mean ± SD)	54 ± 13	54 ± 13	55 ± 13	0.794
Arterial hypertension n (%)	333 (54.0)	183 (54.0)	150 (53.0)	0.943
Diabetes mellitus n (%)	119 (19.0)	65 (19.0)	54 (19.0)	0.961
Hyperlipidemia n (%)	280 (45.0)	144 (42.0)	136 (48.0)	0.124
Atrial fibrillation n (%)	152 (24.0)	81 (24.0)	71 (25.0)	0.662
History of smoking n (%)	292 (47.0)	144 (42.0)	148 (53.0)	0.009*
Familiar DCM n (%)	44 (7.1)	31 (9.1)	13 (4.6)	0.031
ICD implanted before/during hospitalization n (%)	54 (8.7)	30 (8.8)	24 (8.5)	0.910
ICD implanted any time at follow-up n (%)	111 (17.8)	67 (19.6)	44 (15.7)	0.196
CRT implanted before/during hospitalization n (%)	26 (4.2)	10 (2.9)	16 (5.7)	0.087
CRT implanted anytime at follow-up n (%)	90 (14.5)	55 (16.1)	35 (12.5)	0.195
NYHA 1/2 n (%)	301 (48.0)	178 (52.0)	123 (44.0)	0.036*
NYHA 3/4 n (%)	321 (52.0)	163 (48.0)	158 (56.0)	
SBP mmHg (mean ± SD)	121 ± 17	121 ± 17	121 ± 18	0.784
DBP mmHg (mean ± SD)	77 ± 11	76 ± 11	79 ± 12	0.002*
Heart rate bpm (mean ± SD)	80 ± 16	80 ± 16	80 ± 17	0.677
Height cm (mean ± SD)	175 ± 9	174 ± 9	175 ± 10	0.250
Weight kg (mean ± SD)	88 ± 20	88 ± 20	89 ± 20	0.628
BMI kg/m^2^ (mean ± SD)	29 ± 6	29 ± 6	29 ± 6	0.889
Chol mmol/L (mean ± SD)	4.5 ± 1.3	4.5 ± 1.3	4.5 ± 1.2	0.838
HDL-C mmol/L (mean ± SD)	1.1 ± 0.4	1.1 ± 0.4	1.1 ± 0.3	0.858
TG mmol/L (mean ± SD)	1.6 ± 1.1	1.6 ± 1	1.6 ± 1.2	0.858
LDL mmol/L (mean ± SD)	3.5 ± 1	3.9 ± 0,9	3.2 ± 1	0.001*
Sodium mmol/L (mean ± SD)	140 ± 3	140 ± 3	139 ± 3	0.001*
Urea mmol/L (mean ± SD)	6.9 ± 2.9	6.7 ± 2.6	7.1 ± 3.3	0.166
Creatinine μmol/L (mean ± SD)	92 ± 44	89 ± 25	96 ± 59	0.067
Troponin ng/L (mean ± SD)	30 ± 75	25 ± 48	34 ± 95	0.269
NT-proBNP ng/L (mean ± SD)	3057 ± 4355	3017 ± 4171	3103 ± 4567	0.809
BIL-T μmol/L (mean ± SD)	15.6 ± 12.2	15.5 ± 12.4	15.7 ± 11.9	0.819
ALT µkat/L (mean ± SD)	0.71 ± 1.44	0.67 ± 1.3	0.76 ± 1.59	0.458
AST µkat/L (mean ± SD)	0.53 ± 0.9	0.5 ± 0.3	0.58 ± 1.29	0.286
CKD-EPI (mL/s/1.73 m^2^) (mean ± SD)	1.36 ± 0.35	1.37 ± 0.33	1.35 ± 0.38	0.598
HGB g/L (mean ± SD)	144 ± 17	144 ± 16	145 ± 17	0.383
PLT ×10⁹/L (mean ± SD)	208 ± 66	207 ± 67	209 ± 65	0.676
WBC ×10⁹/L (mean ± SD)	7.4 ± 2.3	7.4 ± 2.1	7.3 ± 2.5	0.737
RV mm (mean ± SD)	35 ± 7	34 ± 6	36 ± 7	0.001*
LVEDD mm (mean ± SD)	67 ± 7	66 ± 7	68 ± 8	0.001*
IVS mm (mean ± SD)	9.8 ± 1.7	9.9 ± 1.7	9.8 ± 1.8	0.850
PWD mm (mean ± SD)	9.7 ± 1.5	9.9 ± 1.4	9.5 ± 1.6	0.002*
LVEF % (mean ± SD)	26 ± 7	26 ± 7	26 ± 8	0.545
TAPSE mm (mean ± SD)	18 ± 5	18 ± 5	19 ± 5	0.992
LA mm (mean ± SD)	47 ± 6	47 ± 6	48 ± 6	0.259
TR none/mild	483 (78.0)	268 (79.0)	215 (77.0)	0.699
TR moderate n (%)	100 (16.0)	51 (15.0)	49 (17.0)	
TR severe n (%)	39 (6.0)	22 (6.0)	17 (6.0)	
MR none/mild	327 (53.0)	181 (53.0)	146 (52.0)	0.834
MR moderate n (%)	197 (32.0)	109 (32.0)	88 (31.0)	
MR severe n (%)	98 (16.0)	51 (15.0)	47 (17.0)	
ACEI/ARB/ARNI n (%)	552 (89.0)	304 (89.0)	248 (88.0)	0.726
BB n (%)	569 (91.0)	312 (91.0)	257 (91.0)	0.987
Betablocker dose (converted to metoprolol) mg (mean ± SD)	58 ± 42	60 ± 43	55 ± 41	0.182
MRA n (%)	560 (90.0)	302 (89.0)	258 (92.0)	0.178
SGLT2i n (%)	99 (15.9)	2 (0.6)	97 (34.5)	0.001*
Furosemide n (%)	543 (87.0)	304 (89.0)	239 (85.0)	0.127
Furosemide dosage mg (mean ± SD)	77 ± 78	72 ± 68	83 ± 89	0.122
CKD stages				
Stage 1 n (%)	232 (38.0)	128 (38.0)	104 (38.0)	0.414
Stage 2 n (%)	288 (47.0)	165 (49.0)	123 (44.0)	
Stage 3a n (%)	68 (11.0)	35 (10.0)	33 (12.0)	
Stage 3b n (%)	19 (3.0)	8 (2.0)	11 (4.0)	
Stage 4/5 n (%)	9 (1.0)	3 (1.0)	6 (2.0)	

The mean age at enrollment was 54 ± 13 years, with no significant difference between the two time periods. However, men were significantly younger than women (p = 0.003). A higher proportion of patients in the second era had a history of smoking (53.0% vs. 42.0%, p = 0.009), while familiar DCM was more prevalent among those referred in the first era (9.1% vs. 4.6%, p = 0.031). Overall, men were more likely to have atrial fibrillation than women (p = 0.016) and were also more likely to be smokers (p = 0.001). In the second era, a greater proportion of patients were classified as NYHA class III/IV (56.0% vs. 48.0%, p = 0.036). Additionally, patients from the second era had lower LDL cholesterol levels (3.2 ± 1.0 vs. 3.9 ± 0.9 mmol/L, p = 0.001) and lower serum sodium concentrations (139 ± 3 vs. 140 ± 3 mmol/L, p = 0.001). Echocardiographic measurements showed a larger left ventricular end-diastolic diameter (LVEDD) (68 ± 8 vs. 66 ± 7 mm, p = 0.001) and right ventricular (RV) diameter (36 ± 7 vs. 34 ± 6 mm, p = 0.001) in patients from the second era. There was no statistically significant difference in left ventricular ejection fraction (LVEF) between the two time periods.

All patients received optimal medical therapy in accordance with contemporary international guidelines relevant to the time of treatment. More than 85% of patients were prescribed a combination of angiotensin-converting enzyme inhibitor (ACEI), angiotensin receptor blocker (ARB), or angiotensin receptor/neprilysin inhibitor (ARNI), plus a beta-blocker (BB) and a mineralocorticoid receptor antagonist (MRA). In the second era, a significantly higher proportion of patients were treated with SGLT2i (0.6% vs. 34.5%, p < 0.001). Within the study cohort, 21 patients underwent HTx and 21 received an MCS. All HTx and MCS procedures were performed in cases of severe HF refractory to optimal medical therapy.

Long-term outcome

**Figure 1:**
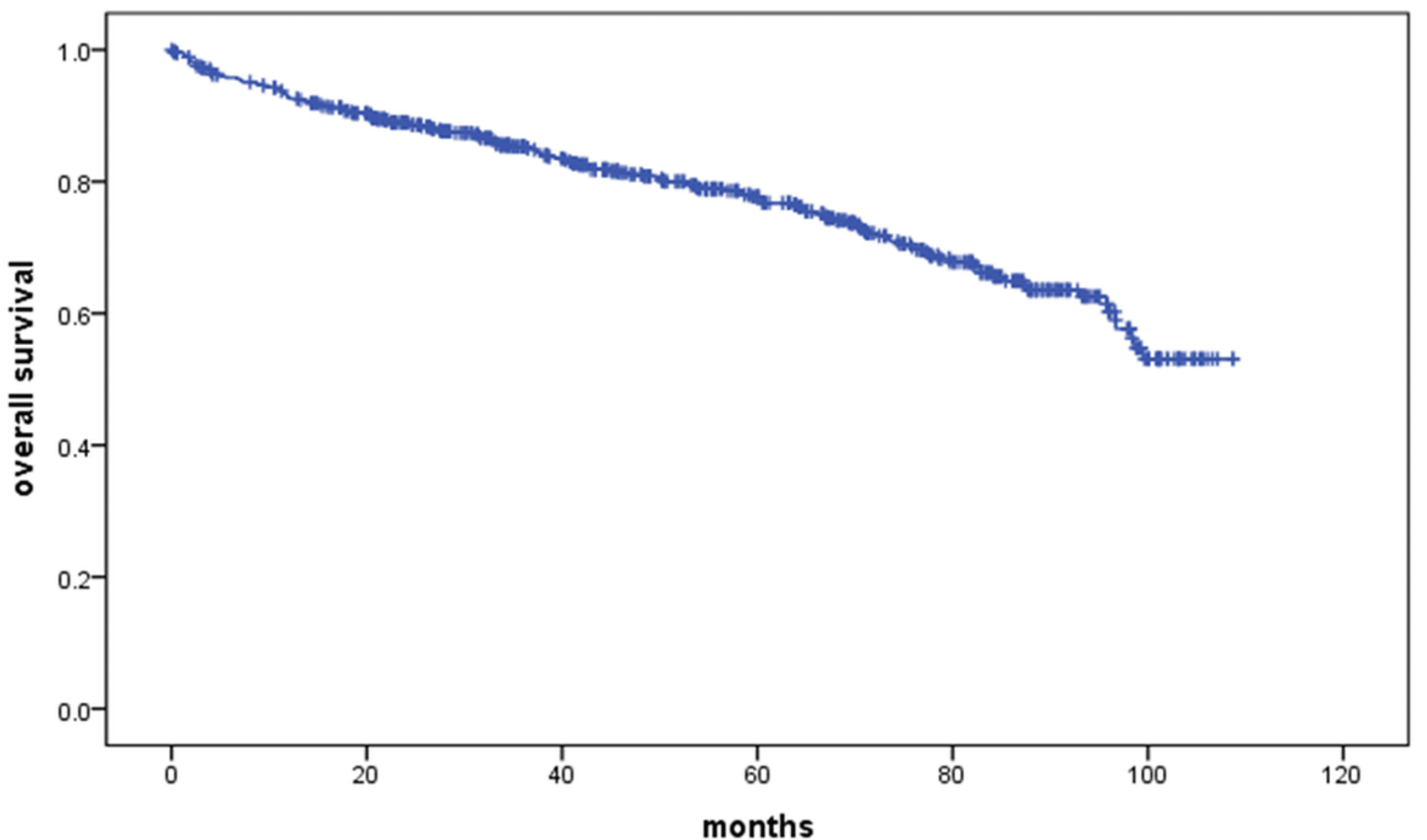
Kaplan-Meier curve for the overall study population

The total cohort included 622 patients, of whom 155 (24.9%) died during the observation period. The remaining 467 patients (75.1%) were censored. Five-year mortality was 22.3%. Figure 1 illustrates the overall survival of the entire cohort of patients from the time of hospitalization. Mean survival was 84.1 ± 1.63 months (95% CI, 80.97-87.37). The "number at risk" displayed in Figure 1 corresponds to the values in Table 2.

**Table 2:** Number of patients at risk at specified time points (corresponding to Figure 1)

Months	0	12	24	36	48	60	72	84	96	108
No. at risk	622	555	472	383	312	253	178	114	51	2

**Figure 2:**
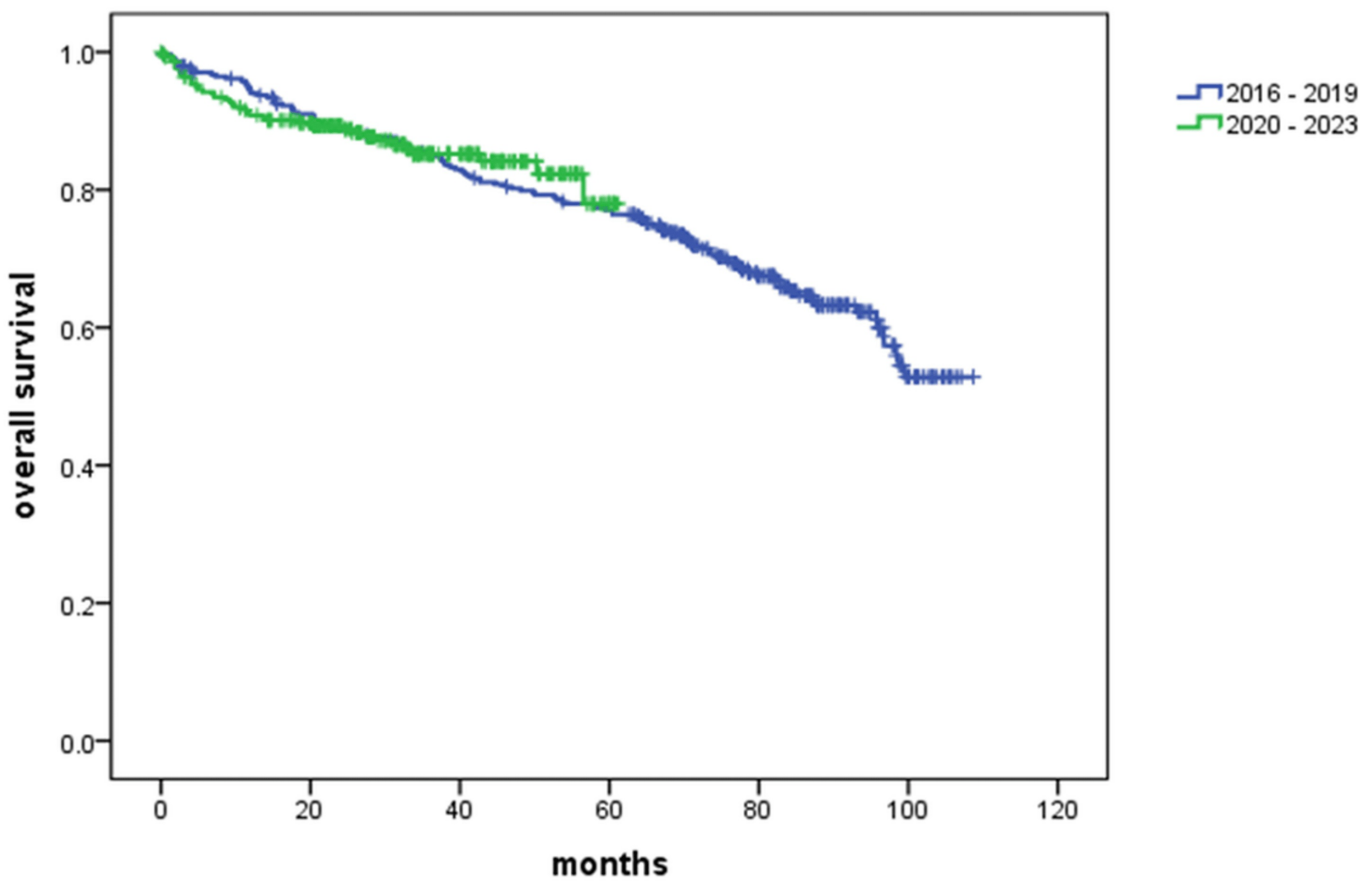
Kaplan-Meier curve for all-cause mortality according to enrollment period

Kaplan-Meier survival curves for patients with DCM stratified by period of hospitalization are shown in Figure 2. The blue curve represents patients hospitalized between January 1, 2016, and December 31, 2019, while the green curve represents patients hospitalized between January 1, 2020, and December 31, 2023. Survival outcomes were comparable between the two groups (p = not significant (NS)), with no statistically significant difference in survival between the two periods. The number at risk shown in Figure 2 corresponds to the data presented in Table 3.

**Table 3:** Number of patients at risk at specified time points (corresponding to Figure 2)

Months	0	12	24	36	48	60	72	84	96	108
2016-2019	341	312	290	276	257	246	178	114	51	2
2020-2023	281	243	182	107	55	7	0	0	0	0

**Figure 3:**
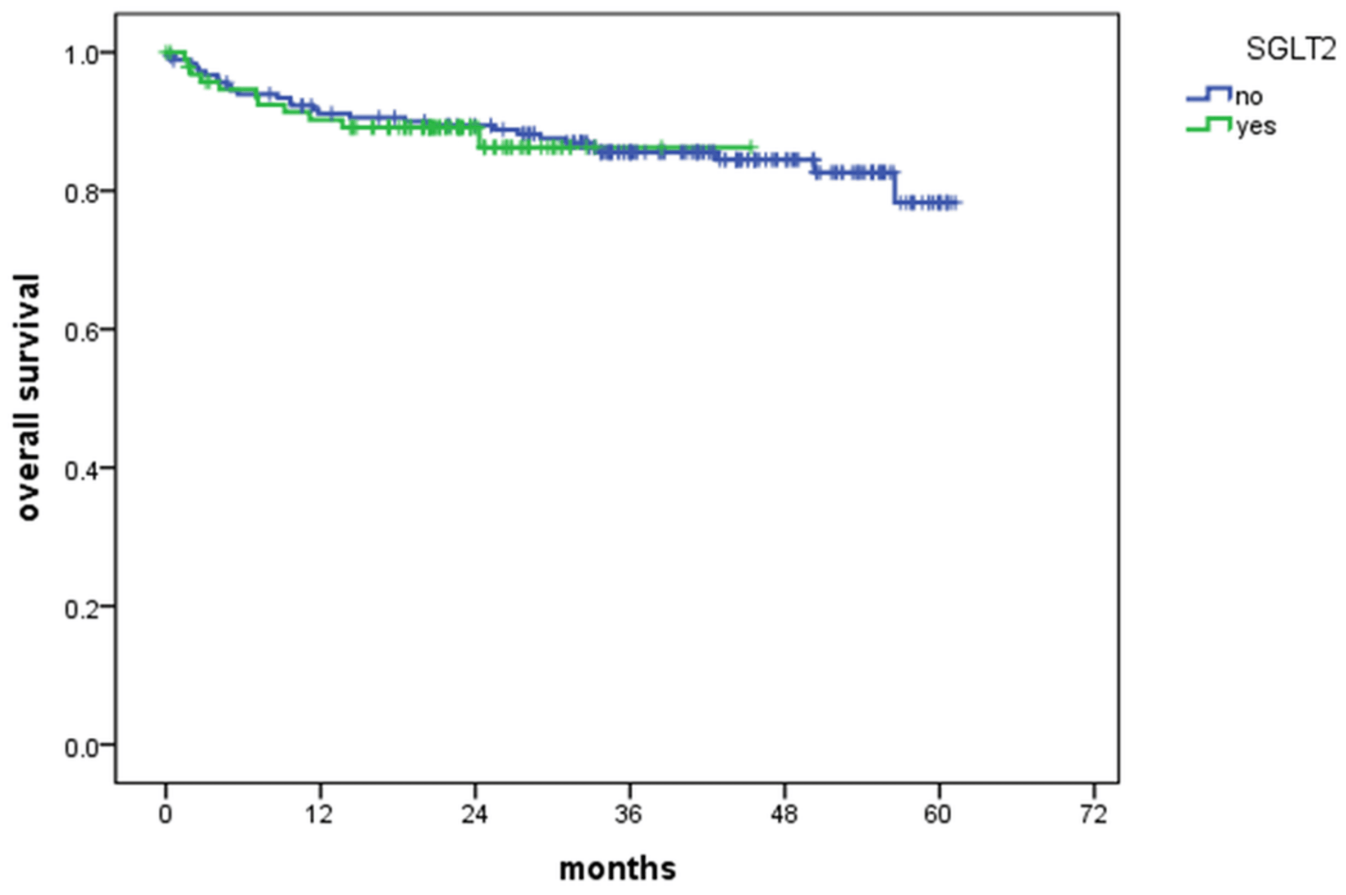
Kaplan-Meier survival curve in patients with and without sodium-glucose cotransporter-2 inhibitors (SGLT2i)

We analyzed the impact of SGLT2i on survival outcomes. Figure 3 shows the survival curve comparing patients treated with SGLT2i to those who were not. No significant difference in survival was observed between the two groups (p = NS). The data labeled "number at risk" under Figure 3 is identical to that provided in Table 4.

**Table 4:** Number of patients at risk at specified time points (corresponding to Figure 3) SGLT2i - sodium-glucose cotransporter-2 inhibitors

Months	0	12	24	36	48	60	72
SGLT2i - no	184	161	149	105	55	7	0
SGLT2i - yes	97	82	33	2	0	0	0

Subgroup analyses

A total of 622 patients were included in the univariate analysis, with restrictions for NT-proBNP (n = 598) and chronic kidney disease (CKD) (n = 616). The final multivariate analysis included 594 patients. A detailed subgroup survival analysis is presented in Table 5.

**Table 5:** Subgroup analysis of cohort ACEI - angiotensin-converting enzyme inhibitor; AF - atrial fibrillation; ARNI - angiotensin receptor/neprilysin inhibitor; BMI - body mass index; CKD - chronic kidney disease; DBP - diastolic blood pressure; DM - diabetes mellitus; LVEF - left ventricular ejection fraction; LVEDD - left ventricular end diastolic diameter; NS - non-significant; NT-proBNP - N-terminal pro-B-type natriuretic peptide; NYHA - New York Heart Association; SBP - systolic blood pressure; % Cens. - percentage of patients without the event during follow-up who were censored at last contact *The level of statistical significance was set at α = 0.05. Mean and median survival times were estimated using the Kaplan-Meier method, with 95% confidence intervals provided for each group. Comparisons between subgroups were evaluated using the log-rank test.

Parameter	Comparison	Patients group A	Events A	% Cens. A	Patients group B	Events B	% Cens. B	Result	p-value
Age (years)	≥60 vs. <60	365	76	79.2%	257	79	69.3%	Poorer survival in older patients	0.011
Sex	Women vs. men	484	128	73.6%	138	27	80.4%	Better survival in women	0.017
BMI (kg/m²)	Obese vs. normal weight	393	108	72.5%	229	47	79.5%	Better survival in obese patients	0.013
Smoking history	Yes vs. no	330	81	75.5%	292	74	74.7%	No difference	NS
NYHA class	III/IV vs. I/II	301	65	78.4%	321	90	72.0%	Poorer survival in NYHA III/IV	0.002
LVEF (%)	<25% vs. >40%	269	81	69.9%	37	5	86.5%	Worse survival with LVEF <25%	0.002
LVEF (%)	25-39% vs. >40%	316	69	78.2%	37	5	86.5%	Worse survival with LVEF 25-39%	0.022
SBP (mmHg)	≥130 vs. <130	408	104	74.6%	214	51	76.2%	No difference	NS
DBP (mmHg)	<70 vs. ≥70	201	56	72.1%	421	99	76.5%	No difference	NS
LVEDD (mm)	<65 vs. ≥65	277	62	77.6%	345	93	73.0%	Trend only	0.051
Heart rate (bpm)	≥90 vs. <90	483	122	74.7%	139	33	76.3%	No difference	NS
CKD (LVEF <40%)	Stages 1-2 vs. stages 3-5	490	112	77.1%	89	37	58.4%	Better survival in stage 1-2	0.001
DM (LVEF <40%)	Without vs. with DM	476	114	76.0%	110	36	67.3%	Better survival in patients without DM	0.027
CKD + DM (LVEF <40%)	With vs. without (CKD 3+)	65	25	61.5%	24	12	50.0%	No difference	NS
CKD (DM + LVEF <40%)	Stages 1-2 vs. stages 3-5	85	23	72.9%	24	12	50.0%	Better survival in stage 1-2	0.016
Atrial fibrillation	AF vs. sinus rhythm	470	105	77.7%	152	50	67.1%	Worse survival in AF	0.002
ACEI vs. ARNI	ACEI vs. ARNI	302	93	69.2%	233	36	84.5%	No difference	NS
NT-proBNP (ng/L)	≤3000 vs. >3000	422	76	82.0%	176	70	60.2%	Worse survival with NT-proBNP > 3000	0.001

We performed Cox regression analysis to identify independent predictors of survival in patients with DCM. The results are presented in Table 6. Negative risk factors associated with worsening survival included age (HR = 1.024, 95% CI: 1.008-1.040, p = 0.003), NYHA III/IV (HR = 1.573, 95% CI: 1.080-2.289, p = 0.018), CKD stages 3-5 (HR = 1.576, 95% CI: 1.021-2.434, p = 0.040), NT-proBNP > 3000 ng/L (HR = 2.020, 95% CI: 1.396-2.922, p < 0.001), and DM (HR = 1.067, 95% CI: 1.076-2.399).

**Table 6:** Multivariable analysis CKD - chronic kidney disease; LVEF - left ventricular ejection fraction; NT-proBNP - N-terminal pro-B-type natriuretic peptide; NYHA - New York Heart Association *Univariate and multivariate Cox proportional hazards model (hazard ratio, HR) was used to identify independent prognostic factors. The univariate and multivariate Cox proportional hazards regression models were performed to identify independent risk factors for patients. HR for age and LVEF represents the risk per one-year and per 1% change, respectively. NT-proBNP dichotomized at 3000 ng/L.

Variable	HR (95% CI)	p-value
Age	1.024 (1.008-1.040)	0.003
Female	0.441 (0.278-0.699)	0.001
Overweight	0.627 (0.420-0.937)	0.023
NYHA 3/4	1.573 (1.080-2.289)	0.018
CKD ≥ stage 3	1.576 (1.021-2.434)	0.040
LVEF	0.972 (0.945-0.999)	0.041
NT-proBNP > 3000 ng/L	2.020 (1.396-2.922)	0.001
Diabetes mellitus	1.607 (1.076-2.399)	0.021

Positive risk factors associated with improved survival included female sex (HR = 0.441, 95% CI: 0.278-0.699, p = 0.001), overweight status (HR = 0.627, 95% CI: 0.420-0.937, p = 0.023), and left ventricular ejection fraction (LVEF) (HR = 0.972, 95% CI: 0.945-0.999, p = 0.041). Factors with no significant impact on survival included obesity, NYHA IV, LVEDD > 65 mm, and a history of atrial fibrillation.

## Discussion

This retrospective study presents a survival analysis of patients with DCM at a single tertiary center. Our findings indicate a five-year mortality rate of 22.3% and a nine-year mortality rate of 46.9%. While the prognosis for patients with DCM has improved over the years, effective risk stratification remains crucial throughout the course of the disease. Our data suggest better survival rates compared to previous studies. The EPICAL study reported a five-year mortality rate of 65.5% among patients with HF with reduced ejection fraction (HFrEF). Several factors may explain the discrepancy between our findings and those of the EPICAL study. The mean age of patients in our cohort was lower (54 vs. 64 years), and the EPICAL study included patients with a broader range of HF etiologies. [13]. Another study focusing only on DCM patients reported a five-year mortality of 55.9% [14]. In addition, our study identified age as an independent prognostic risk factor affecting survival in DCM patients. Aging is associated with progressive deterioration in cardiac function and an increased burden of comorbidities.

At a period of approximately 96 months, we observed a sudden decline in overall survival. This phenomenon likely reflects the natural course of the disease in patients hospitalized at the beginning of the follow-up period. It is probable that this group consisted of patients with longer disease duration, who experienced a gradual deterioration in clinical condition leading to death over time. Additionally, some of these patients may have been affected by a COVID-19 infection, which could have further contributed to the worsening of their health. One possible explanation for these differences in survival is the evolution of HF management over time when treatment strategies were less advanced, particularly regarding pharmacotherapy and device-based interventions.

The NYHA classification is a widely used system to classify patients with HF. According to our results, patients with NYHA III/IV had poorer survival compared to patients with NYHA I/II. Numerous studies have conducted research about the prognostic role of the NYHA classification in HF, with different results. The Briongos-Figueroa et al. study found that a worse NYHA class independently increases the risk of cardiovascular death [15]. Multivariate analysis confirmed that NYHA class III/IV was an independent predictor of worse prognosis. On the other hand, a meta-analysis from 2019 found that NYHA classification is an unreliable predictor of adverse outcomes in HF [16]. Given these results, we believe that mortality should be assessed comprehensively, rather than relying on a single parameter. Additionally, female sex was identified as a positive independent risk factor. This finding is consistent with several studies that have shown women tend to have better outcomes compared to men in DCM [17].

The widespread implementation of ICD, CRT, and adherence to guideline-directed medical therapy (GDMT) have likely contributed to improved survival outcomes. Merlo et. al [18] demonstrated that the incidence of major cardiac events was reduced at a later stage of disease following implantation of ICD or CRT. Long-term prognosis has also been shown to improve due to advancements in pharmacological therapies and device-based management [19]. A patient’s age also plays a significant role in survival outcomes. A study from 2012 reported that for every 10-year increase in age, the risk of five-year mortality rises by 31% [20].

In this analysis, we compared two time periods: 2016-2019 and 2020-2023. These periods were selected to assess potential influences of the COVID-19 pandemic (2019-2022) and the introduction of SGLT2i in 2022. However, no significant difference in survival was observed between those two periods, nor was there a difference in survival between patients who received SGLT2i and those who did not.

The number of patients enrolled in the second era was lower than in the first, which could be attributed to the impact of the COVID-19 pandemic on healthcare access and hospital admissions. Additionally, patients with a stable DCM may have been managed by district cardiologists and referred to the tertiary care centers only upon disease progression. These findings suggest that despite advancements in HF treatment, long-term survival in our DCM cohort has not significantly improved. Potential contributing factors include stabilization of treatment strategies.

Among the patients included in this study, those with obesity showed better survival outcomes than those with a normal BMI. The so-called obesity paradox has been described in patients with HF, suggesting that obese individuals may have a better prognosis than those with a normal BMI [21]. However, this association was not confirmed in our multivariate analysis.

Interestingly, overweight status (BMI 25-30 kg/m²) was identified as an independent negative prognostic factor. This finding suggests that the initial observation may have been influenced by confounding factors. However, this result should not be dismissed entirely, as obesity may contribute to the development of DCM through mechanisms such as stimulation of collagen synthesis mediated by immune responses and ferroptosis, which is associated with increased oxidative stress [22].

Patients with NT-proBNP > 3000 ng/mL had worse survival than those with lower NT-proBNP levels. Multivariate analysis confirmed that NT-proBNP > 3000 ng/mL is an independent risk factor affecting survival. These findings align with previous research, such as the study by Aaronson et al [23], which demonstrated that patients with NT-proBNP levels below 4500 pg/mL had better event-free survival. This highlights the prognostic value of NT-proBNP in the risk stratification of patients with DCM.

HF patients often suffer not only from the condition itself but also from coexisting diseases such as DM and CKD. The combination of these comorbidities is associated with a higher risk of adverse outcomes [24]. The coexistence of these conditions is now commonly referred to as the cardio-renal-metabolic (CRM) continuum. A 2022 study reported that the adjusted risk of mortality in patients with HF and CKD was increased six- to seven-fold [25]. Our results confirm these previous studies. According to multivariable analysis, both DM and CKD stages 3-5 were independent risk factors affecting survival. To date, the most extensive analysis of the impact of CRM has come from the DELIVER study, which primarily focused on patients with HF with preserved ejection fraction (HFpEF) [26]. Nevertheless, multimorbidity within the CRM spectrum is also associated with a markedly increased risk in patients with HFrEF. Therefore, emphasis should be placed on the management of individual risk factors, multidisciplinary collaboration, the use of novel therapies such as SGLT2i, and a comprehensive, patient-centered approach to care.

According to the latest ESC recommendations for the treatment of HF [1], optimal HF treatment should include four key pharmacological groups: ACEI/ARNI/ARBs, BB, MRA, and SGLT2i. In our study, more than 85% were treated with ACEI/ARNI/ARBs, MRA, and BB, and just 34.5% patients were treated with SGLT2i. This is significantly higher than reported in the CHAMP-HF study, where 27% of patients were not on ARNI/ACEI/ARBs, 33% were not receiving a BB, and 67% of patients were not treated with MRAs [27].

One potential reason for the lower SGLT2i usage in our cohort is the relatively recent introduction of these agents. In Slovakia, SGLT2i therapy for HFrEF was officially categorized on May 15, 2022, and our SGLT2i group included only 97 patients. Hospitalization at our tertiary center may have contributed to a higher proportion of patients receiving guideline-recommended therapy. The most common reason for not prescribing these treatments was medication intolerance.

Assessment of cardiac function most commonly relies on LVEF, which also provides valuable prognostic information in HF. According to PARADIGM-HF, the risk of all outcomes increased with decreasing LVEF. Each five-point reduction in LVEF was associated with 9% increased risk of cardiovascular death [28]. Results of our multivariate analysis suggest that each 5% increase in LVEF is associated with a 14% lower risk of death.

The primary limitation of this study is its retrospective design. The accuracy of the data relies on the quality of medical records, which may be incomplete and lack standardization. Additionally, echocardiographic parameters can vary depending on the examiner, introducing potential inconsistencies. The absence of patient randomization may also lead to selection bias. The study population was ethnically homogeneous, reflecting the population of Slovakia, which may limit the generalizability of the findings to more diverse populations. Furthermore, incomplete data on treatment adherence and socioeconomic factors may have affected the results. However, this study represents a real-life, unselected, consecutive patient cohort, offering valuable insights into the survival of contemporary DCM patients from a single tertiary center in Slovakia. The comparison of survival between the two time periods may be affected by the shorter follow-up duration in the 2020-2023 cohort, which could have limited the ability to detect potential differences in long-term outcomes.

## Conclusions

This retrospective study analyzed survival in patients with DCM treated at a single tertiary center. Our data suggest better survival rates compared to historical cohorts, likely reflecting advances in HF management and widespread use of guideline-directed therapy. Although long-term survival remained stable across the two analyzed periods, this may partly result from differences in follow-up duration between the compared eras. This study is unique in providing real-world insights into the management and outcomes of patients with DCM from a tertiary center. These results may serve as a basis for future studies at a national level, which could provide even more comprehensive insights into the management and long-term outcomes of DCM patients.
